# Molecular signatures distinguish senescent cells from inflammatory cells in aged mouse callus stromal cells

**DOI:** 10.3389/fendo.2023.1090049

**Published:** 2023-02-16

**Authors:** Jiatong Liu, Xi Lin, Andrew McDavid, Yutiancheng Yang, Hengwei Zhang, Brendan F. Boyce, Lianping Xing

**Affiliations:** ^1^ Department of Pathology and Laboratory Medicine, University of Rochester Medical Center, Rochester, NY, United States; ^2^ Biostatistics and Computational Biology, University of Rochester Medical Center, Rochester, NY, United States; ^3^ Orthopaedics and Rehabilitation, Center for Musculoskeletal Research, University of Rochester Medical Center, Rochester, NY, United States

**Keywords:** inflammation, ScRNA-seq, aging, fracture, senescence

## Abstract

Cellular senescence plays important roles in age-related diseases, including musculoskeletal disorders. Senescent cells (SCs) exert a senescence-associated secretory phenotype (SASP) by producing SASP factors, some of which overlap with factors produced by inflammatory cells (Inf-Cs). However, the differences between SCs and Inf-Cs and how they interact with each other during fracture repair have not been well studied. Here, we analyzed single cell RNA sequencing data of aged mouse fracture callus stromal cells. We defined Inf-Cs as cells that express NF-κB *Rela/Relb*, SCs as cells that express the senescence genes, *Cdkn1a*, *Cdkn2a* or *Cdkn2c*, and inflammatory SCs (Inf-SCs) as cells that express both NF-κB and senescence genes. Differentially expressed genes and pathway analyses revealed that Inf-SCs and SCs had a similar gene expression profile and upregulated pathways that are related to DNA damage/oxidation-reduction and cellular senescence, while Inf-Cs expressed different gene signatures and pathways from SCs and Inf-SCs, mainly related to inflammation. Cellchat software analysis indicated that SCs and Inf-SCs are potential ligand-producing cells that affect Inf-Cs as target cells. Cell culture experiments demonstrated that SC conditioned medium promoted inflammatory gene expression by callus-derived mesenchymal progenitor cells, and Inf-Cs had reduced osteoblast differentiation capacity. In summary, we have identified three cell subclusters associated with inflammation and senescence in callus stromal cells, predicted potential effects of Inf-SCs and SCs on Inf-Cs by production of active ligands, and demonstrated that when mesenchymal progenitors acquire inflammatory phenotypes their osteogenic potential is reduced.

## Introduction

Cellular senescence plays important roles in human diseases, including musculoskeletal disorders (1, 2). Recently we found that aged mice have markedly increased senescent cells (SCs) in fracture callus that exert the senescence-associated secretory phenotype (SASP). Clearance of SCs with senolytic drugs promotes fracture healing in aged mice (3) and young mice (4). However, fractures are also associated with inflammation. Inflammatory cells (Inf-Cs) produce pro-inflammatory factors, some of which overlap with SASP factors (5, 6).

Pre-clinic studies have revealed potential difference between SCs and Inf-Cs in fracture healing. For instance, the acute inflammatory response in the callus following fracture peaks at 24-48 hours and quickly disappears within a week, and deficiency or inhibition of inflammation at this stage causes delayed fracture healing by inhibiting endochondral ossification (7, 8). In contrast, SCs accumulate in the callus gradually and peaks around 10-14 days post-fracture and removing SCs by senolytic drugs, given at 3-7 days post-fracture before the peak of SC accumulation, improves fracture healing (3).

Distinguishing SCs from Inf-Cs in aged callus is very important, not only because both of them are increased and produce inflammatory factors, but also because they require different drug treatments, e.g. senolytic drugs for SCs, and non-steroidal anti-inflammation drugs (NSAIDs) for Inf-Cs. NSAIDs improve fracture healing in mice (9), but the drugs have been reported to significantly increase the risk of a second hip fracture, especially in old patients (10). The meta-analysis of the NSAID treatment in clinical trials reports a negative effect of NSAID, which is highly dose and time dependent because long term NSAID administration increase the rates of non-union fracture in the elder patients (11, 12). These studies indicate that anti-inflammatory drugs may not be a good choice for treating fractures in the elderly.

Thus, identification of any differences between the behavior of Inf-Cs and SCs for treatment of aging fracture is an important unmet need. Here, we analyzed our recently published single cell RNA-sequencing (scRNA-seq) data set of aged mouse callus stromal cells (13). We compared cells that express inflammatory genes and cells that express senescence-associated genes to study molecular signatures of Inf-Cs and SCs and their interactions using bioinformatic analyses. We validated our findings with cell culture experiments and callus cells from NF-κB-GFP reporter mice.

## Materials and methods

### Analysis of single cell RNA-sequencing dataset

scRNA-seq data that we published recently were reanalyzed using cells from aged callus (13) (https://www.ncbi.nlm.nih.gov/geo/query/acc.cgi?acc=GSE199755, GEO, GSE199755). In our previous study, we collected CD45^-^CD31^-^Ter119^-^ stromal cells from callus of 4-month-old young (equivalent to a 26-year-old human) and 21-month-old (equivalent to a 62-year-old human) aged C57BL/6J male mice at 10-day post-fracture (dpf) by fluorescence-activated cell scoring (FACS), the time point when the expression levels of senescence-associated genes reach the peak (3). In the current study, we analyzed data from 6,834 aged stromal cells using a Seurat (version 4.0.6) R package. The rationale of using the data from aged mice is that the callus in aged mice significantly higher levels of inflammation and cellular senescence than those in young mice (3). In brief, the top 2,000 variable genes were identified and ranked by coefficient of variation. The reason why only top 2,000 variable genes were identified is that we used the same threshold described in the original study for Seurat packages (14), where Stuart et al. used only top 2,000 variable genes in their analysis and concluded that focusing on these high variable genes in downstream analysis helps to highlight biological signal in single-cell datasets. Dimensionality reduction of datasets was performed by “RunPCA” function with 10 principal components (npcs = 25) at a resolution of 0.1. Find Neighbors function was used to compute the shared nearest-neighbors for a given dataset with parameter k = 25. Clusters of cells were identified based on SNN modularity optimization with “FindCluster” function. “RunUMAP” function was further used to perform Uniform Manifold Approximation and Projection (UMAP) dimensional reduction. Cell clusters were visualized on reduced UMAP dimensions using “DimPlot” function. Differentially expressed genes (DEGs) of each cluster were identified with “FindAllMarkers” function. The top 10 DEGs with the highest average log 2-fold-change were presented in a heatmap using “DoHeatmap” function for cluster functional annotation.

#### Functional enrichment analysis

To examine the biological processes and signaling pathways in various cell subsets, we performed Gene Ontology (GO) and Kyoto Encyclopedia of Genes and Genomes (KEGG) enrichment analyses. DEGs identified in Seurat/R and with an average log 2-fold-change>0.414, p value<0.05 were used. The top 6 upregulated genes corresponding to biological process or pathways with the highest –log 10-fold-change were presented and used for cluster functional annotation.

#### Cell-cell commination analyses

To identify and illustrate intercellular signaling communication, we used an open-source R package iTALK (http://github.com/Coolgenome/iTALK) that is designed to profile and visualize the ligand-receptor mediated inter-cellular cross-talk signal using scRNA-seq data. In brief, we converted Seurat object to Cellchat/R object, a publicly available database of 2021 with validated molecular interactions for Mus Musculus for ligand-receptor identification (15). We inferred the communication probability/strength between interaction groups using computeCommunProb/CellChat/R function with “type=truncateMean” as the gene expression average method and “trim=0.1” as the threshold. We set Inf-SCs and SCs as the sender/source cells and Inf-Cs as receiver cells, based on Cellchat predicted result to predict the potential ligand-receptor pairs. We also applied the Nichenet packages, a method that predicts ligand- downstream target genes between interacting cells by combining their gene expression data with prior knowledge on signaling and gene regulatory networks (16). Similar to the Cellchat analysis, Inf-SCs and SCs were set as sender/niche cells and Inf-Cs were set as receiver/target cells.

### Validation experiments

#### Animals and tibial fracture procedure

NF-κB-GFP reporter mice (Strain#: 027529) on a C57BL/6 background were purchased from the Jackson Laboratory (17), in which a transgenic construct contains an enhanced GFP-luciferase fusion gene under the control of tandem copies of a 36-base enhancer (containing two NF-κB binding sites) upstream of a herpes simplex virus minimal thymidine kinase promoter. Mice were housed in micro-isolator technique rodent rooms. We used 4-month-old male mice in the current study to exclude the effects of variations in levels of female sex hormones. Open tibial fractures were performed according to the standard operating procedure established in the URMC Center for Musculoskeletal Research (18). In brief, a 5 mm long incision was made in the skin on the anterior side of the tibia after anesthesia. A sterile 27 G needle was inserted into the marrow cavity of the tibia from the proximal end, temporarily withdrawn to facilitate midshaft transection of the tibia using a scalpel, and then reinserted to stabilize the fracture. The incision was closed with 5-0 nylon sutures. Mice received buprenorphine SR, 0.5 mg/kg to control pain. Fractures were confirmed by radiography using a Faxitron device (Hologic, Marlborough, MA). All animal procedures were conducted in accordance with approved guidelines of the University of Rochester Committee for Animal Resources (protocol number: 2001-121R).

#### Callus-derived mesenchymal progenitor cell preparation, cell growth, and osteoblast differentiation assays

For CaMPC preparation, mice were euthanized by CO_2_ inhalation and secondary cervical dislocation at 10 dpf. Surrounding soft tissue was dissected from callus, which was cut into pieces. Callus pieces were washed thoroughly with cold PBS and then digested with ACCUMAX cell detachment solution (Stem cell Tech) for 30 minutes at room temperature and cultured in basal medium (alpha-MEM medium containing 15% FBS). Cells that migrated from callus pieces were cultured to confluence in basal medium and named CaMPCs. For cell growth assays, CaMPCs from NF-κB-GFP mice were subjected to FACS to collect GFP^+^ and GFP^-^ cells. GFP^+^ and GFP^-^ cells were cultured in basal medium for 2 days and stained with a cell counting kit 8 (CCK8) (Abcam, cat#: ab228554) following the manufacturer’s instructions. For osteoblast differentiation assays, GFP^+^ and GFP^-^ cells were cultured in basal medium containing 10% FBS with 50 μg/ml ascorbic acid and 10 mM β-glycerophosphate for 14 days and stained for alkaline phosphatase with 1-step NBT/BCIP reagent (Thermo Scientific, cat#: 34042).

#### RT-qPCR

RNA was extracted in TRIzol solution and cDNA was synthesized using the iSCRIPT cDNA synthesis kit (BioRad). qPCR was performed with iQ SYBR Green Supermix using an iCycler PCR machine (BioRad). *β-actin* was amplified on the same plates and used to normalize the data. Each sample was prepared in triplicate and each experiment was repeated at least once. The relative abundance of each gene was calculated by subtracting the CT value of each sample for an individual gene from the corresponding CT value of *β-actin* (ΔCT). ΔΔCT was obtained by subtracting the ΔCT from the reference point. These values were then raised to the power 2 (2ΔΔCT) to yield fold-expression relative to the reference point. Representative data are presented as means ± SD of the triplicates or of 3 wells of cell cultures. The sequences of primers and qPCR conditions used in current study are shown in **Supplemental Table 1**.

#### Beta-galactosidase activity assay

Senescent cells (SCs) were detected used the fluorescent senescence-associated-β-galactosidase assay (19, 20). Callus cells or cultured CaMPCs were treated with 100 μM 9*H*-(1,3-dichloro-9,9-dimethylacridin-2-one-7-yl) β-D-galactopyranoside (DDAOG) Thermo Fisher Scientific, cat#: D-6488) for 50 minutes and subjected to flow cytometry for 7-hydroxy-9H(I,3-dichloro-9,9-dimethylacridin-2-one) signal.

#### Statistical analysis

Statistical analysis was performed using GraphPad Prism 9 software (GraphPad Software Inc., San Diego, CA, USA). Data are presented as mean ± SD. Levene’s test was used to evaluate the homogeneity of variance and Shapiro Wilk test was used to assess the normality of the data. The *in vitro* data presented in this study are homogenous and fit into normal distribution. Comparisons among 3 or more groups were analyzed using One-way (for groups with one variable) or Two-way (for groups with two variables) ANOVA following Tukey *post-hoc* test. p values <0.05 were considered statistically significant.

## Results

**Figure 1:**
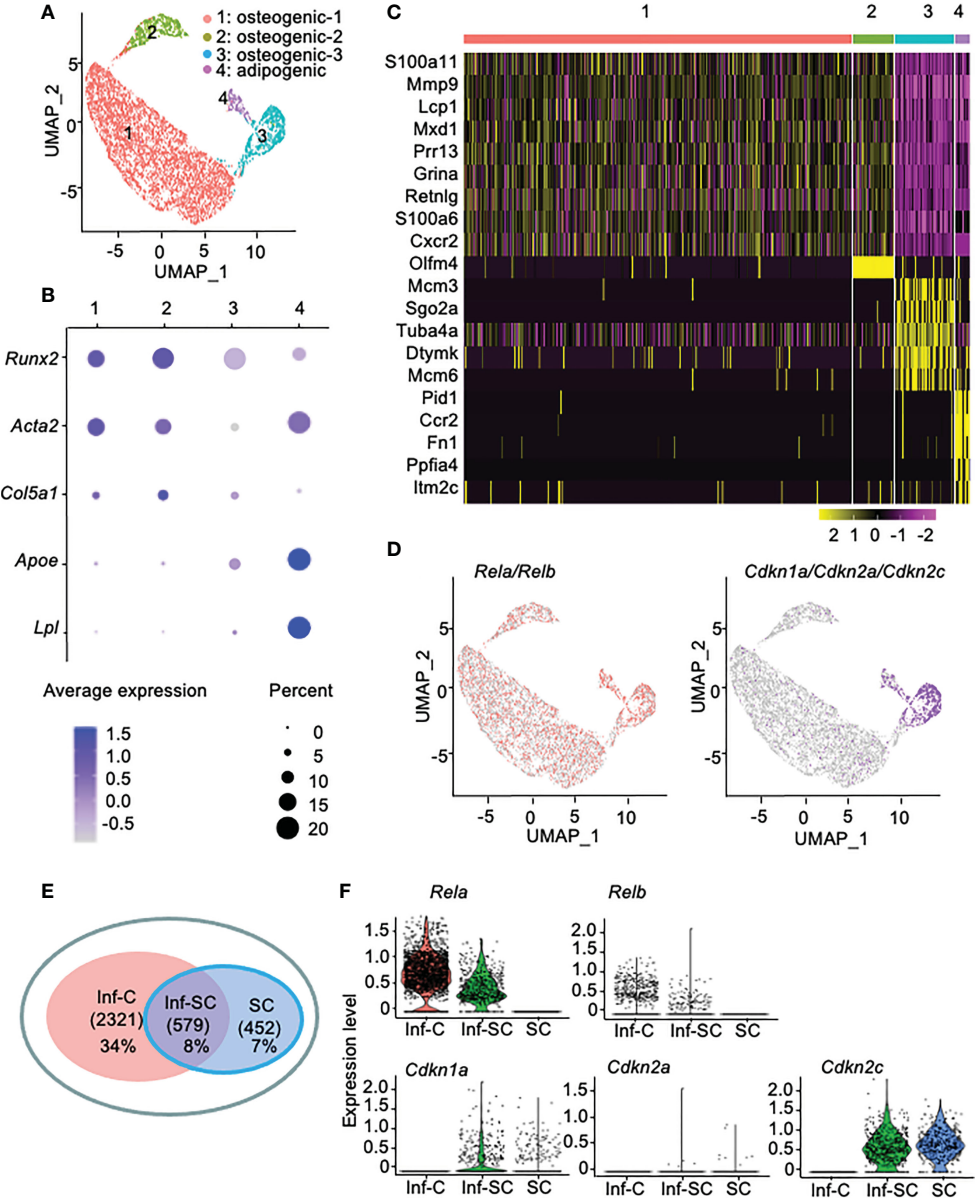
Callus stromal cells contain cell subsets that express genes related to inflammation, senescence, and inflammation/senescence. scRNA-seq dataset from aged (21-month-old) callus CD45^-^CD31^-^Ter119^-^ stromal cells were analyzed. **(A)** A total of 6,834 cells were subjected to unsupervised SNN clustering using Seurat/R and identified 4 major clusters: cluster1: osteogenic cells-1, cluster2: osteogenic cells-2, cluster3: osteogenic cells-3, and cluster4: adipogenic cells. **(B)** Expression of putative osteogenic and adipogenic marker genes: *Runx2* and *Col5a1* (osteoblast-lineage), *Acta2* (skeleton stromal/stem cell-lineage), and *Apoe* and *Lpl* (adipocyte-lineage). **(C)** Heatmap of the top 10 DEGs in clusters1-4 showing cluster1 and cluster2 expressed inflammatory and matrix related genes, cluster3 expressed histone genes, and cluster4 expressed inflammatory and oncogenes. **(D)** Feature plot of expression of inflammatory genes (*Rela* and *Relb*) or senescence-associated genes (*Cdkn1a, Cdkn2a, Cdkn2c*). **(E)** Venn plot of cell numbers in Inf-C (inflammatory+senescent-cells), Inf-SC (inflammatory+senescent+ cells) and SC (inflammatory-senescent+ cells). **(F)** Violin plot of the expression level of inflammatory genes (*Rela* and *Relb*) or senescence-associated genes (*Cdkn1a, Cdkn2a, Cdkn2c*) in three subclusters.

Callus stromal cells contain distinct cell sub-populations that express genes associated with inflammation, senescence, or combined inflammation and senescence. To investigate the differences between callus inflammatory cells (Inf-Cs) and senescent cells (SCs), we analyzed our recently published scRNA-seq dataset of CD45^-^CD31^-^Ter119^-^ callus stromal cells (13). We used data from aged callus cells because inflammation and cellular senescence are increased with aging (13, 21). We identified 4 major cell clusters by graph-based clustering (**Figure 1A**). Cluster1 and cluster2 expressed known osteogenic genes (*Runx2, Acta2, Col5a1*), cluster3 expressed osteogenic genes with a relative lower level than cluster1 and 3, and cluster4 expressed known adipogenic genes (*Apoe, Lpl*) (**Figure 1B**). Thus, we named cluster1, cluster2, cluster3 as osteogenic-1, osteogenic-2, osteogenic-3, and cluster4 as adipogenic cluster, respectively (**Figure 1A**). Heatmap of top differentially expressed genes (DEGs) showed that similar to our previous report that aged callus cells have a high inflammatory signature (13), cells in clusters1, 2 and 4 expressed inflammatory (*S100a11, Retnlg, Mmp9, Cxcr2, and Ccr2*) genes. Interestingly, cells in cluster3 expressed genes associated with chromosome modification and cellular senescence (*Tuba4a and Dtymk*) (**Figure 1C**).

To label inflammatory cells and SCs for further analysis, we defined cells that express the inflammation-related genes, *Rela* or *Relb* (22), as the inflammatory cell (Inf-C) subset and cells that express senescence-associated genes as the SC subset. We examined the expression of 10 genes that have been used to detect SCs of various cell types in the literature (23, 24) and decided to use *Cdkn1a*, *Cdkn2c* and *Cdkn2a* as senescence-associated genes because *Cdkn1a* and *Cdkn2c* are expressed mainly by cluster 3 (**Supplemental Figure 1**) and *Cdkn2a* has been used in our previous study to label callus SCs (3). Thus, we defined cells that express *Cdkn1a*, *Cdkn2a* or *Cdkn2c* as SCs. Here Inf-Cs did not express *Cdkn1a*/*Cdkn2a*/*Cdkn2c*, while SCs did not express *Rela*/*Relb.* Inf-Cs were present in all clusters, while SCs were mainly localized in cluster3, the osteogenic-3, and cluster4, the adipogenic cluster. Some cells in the adipogenic cluster co-expressed both inflammation and senescence-associated genes and were named as the inflammatory SC (Inf-SC) subset (**Figure 1D**). Inf-Cs comprised 34% (2321), Inf-SCs 8% (579), and SCs 7% (452) of the total 6,834 cells analyzed (**Figure 1E**). The expression pattern of these inflammatory and senescence genes in the 3 subsets was illustrated in a violin plot (**Figure 1F**).

**Figure 2:**
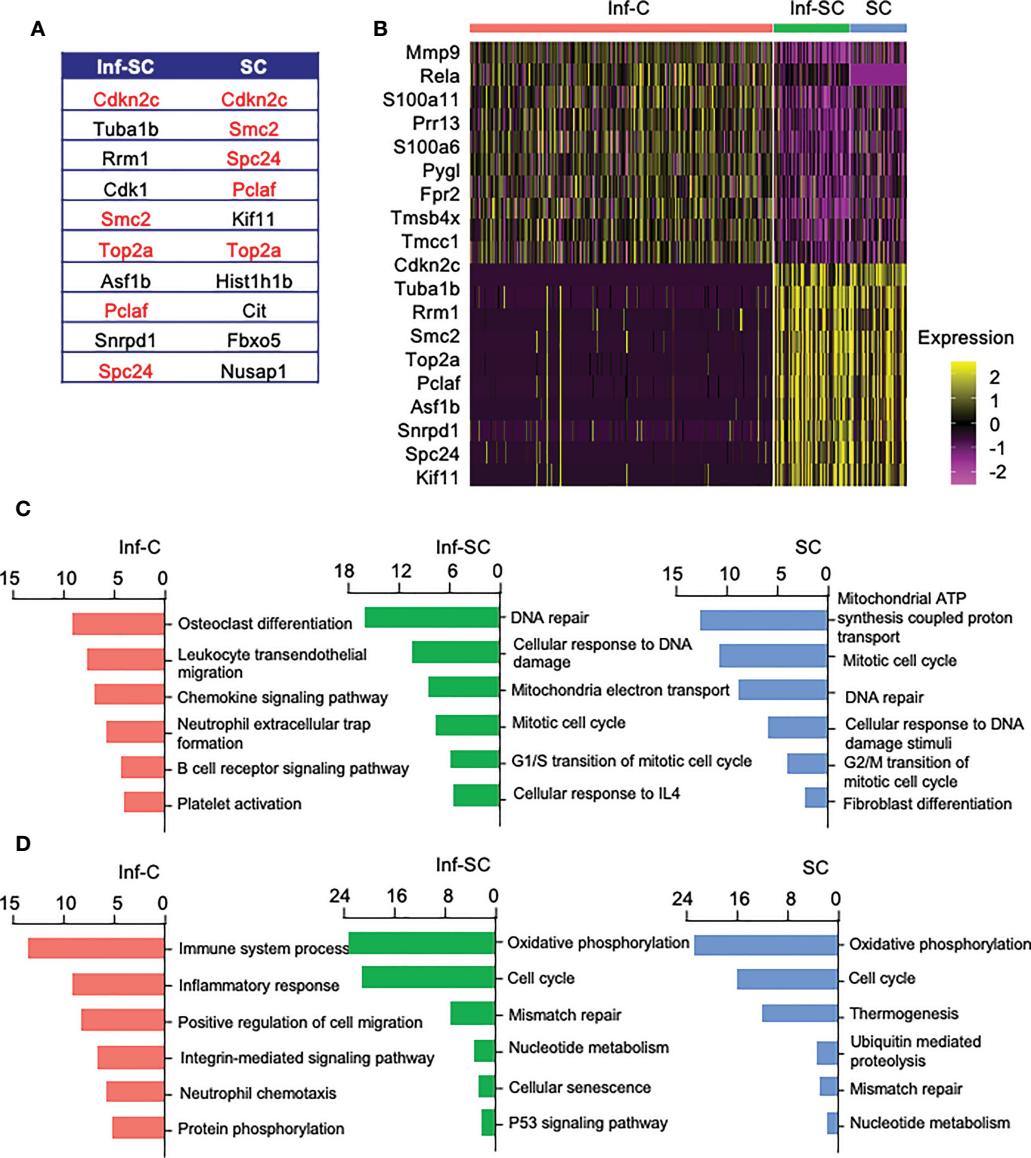
Inflammatory, inflammatory senescent, and senescent cells have distinct signature genes and up-regulated pathways. **(A)** List of top 10 DEGs in Inf-SCs and SCs. Red indicates same signature genes between Inf-SC and SC. **(B)** Heatmap of the top 10 DEGs in Inf-Cs, Inf-SCs and SCs. Top 6 upregulated pathways by GO **(C)** and KEGG **(D)** analysis using the DEGs with Log2Fold changes greater than 25%.

To investigate functional differences among Inf-C, Inf-SC, SC subsets, we compared their top 10 DEGs. A half of the top 10 DEGs in Inf-SCs and SCs overlapped (**Figure 2A**), while Inf-Cs expressed a different set of genes. The heatmap revealed that Inf-Cs highly expressed the matrix degrading gene, *Mmp9*, and other inflammation-associated genes (*S100a6, S100a11*). Inf-SCs and SCs highly expressed *Cdkn2c*, a gene associated with cellular senescence, and *Tuba1b* gene-associated with histone modification (**Figure 2B**). Although ~half of the genes expressed by Inf-SCs and SCs were similar, *Tuba1b*, *Rrm1*, *Cdk1*, *Asf1b* and *Snrpd1* were distinct in Inf-SCs, while *Kif11*, *Hist1hib*, *Cit*, *Fbxo5*, and *Nusap1* were expressed only by SCs (**Figures 2A, B**). Gene ontology (GO) analysis revealed that the top 6 GO terms corresponding to biological process in the Inf-C subset were mainly related to chemokine and immune cell-related signaling pathways, while those in the Inf-SC and SC subsets were mainly related to DNA damage and repair, oxidation-reduction and cellular senescence (**Figure 2C**). KEGG analysis of 6 upregulated pathways also showed a trend similar to GO analysis (**Figure 2D**).

**Figure 3:**
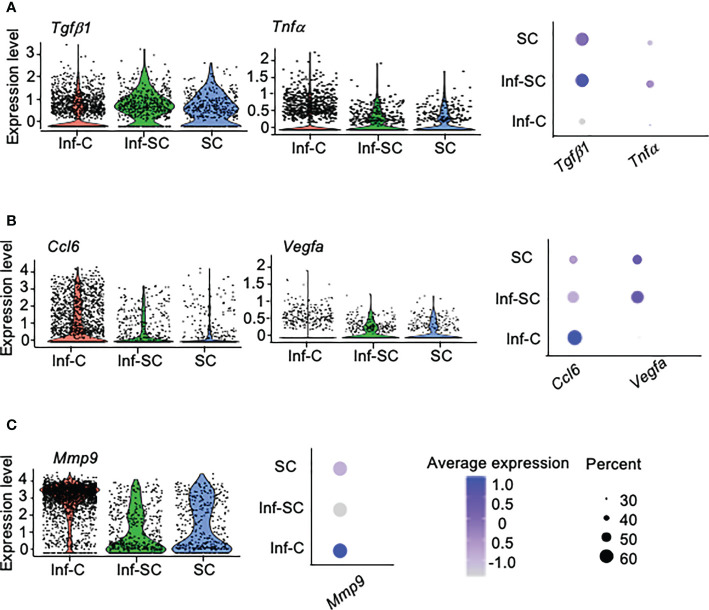
Differentially expressed inflammatory factors among inflammatory, inflammatory senescent and senescent cells. **(A)** Differential expression of SASP factors identified in aged callus tissues. **(B)** Differential expression of cytokines based on the gene list in a mouse cytokine array. **(C)** Differential expression of the pro-inflammatory protease, *Mmp9*, among top 10 DEGs in **Figure 2B**.

SCs acquire senescence-associated secretory phenotype (SASP) (25). We reported higher expression of 12 SASP factors in callus tissue from aged mice than those from young mice, including *Tgfβ1, Tnfα, Il1a, Il1b, Il4, Il6, Csf1, Cxcl1, Cxcl2, Ccl3, Ccl5, and Ccl8* (3). Among these SASP factors, only *Tgfβ1* and *Tnfα* showed differential expression in Inf-C and Inf-SC, SC subsets. *Tgfβ1* and *Tnfα * were expressed mainly by Inf-SC and SC subsets (**Figure 3A**). In addition, we examined the expression of 111 cytokine genes listed in a commercial mouse cytokine array (http://www.rndsystems.com/products/proteome-profiler-mouse-xl-cytokine-array_ary028) and found that *Ccl6* and *Vegfa* had different expression profiles among the 3 cell subsets. *Ccl6* was expressed mainly by the Inf-C and Inf-SC subsets, while *Vegfa* was expressed mainly by the Inf-SC and SC subsets (**Figure 3B**). As the heatmap shows in **Figure 2B**, *Mmp9* was expressed by all 3 subsets, but the expression level was the highest in Inf-Cs (**Figure 3C**).

### Cell-cell communication analyses reveals that inflammatory senescent and senescent cells regulate inflammatory cells

**Figure 4:**
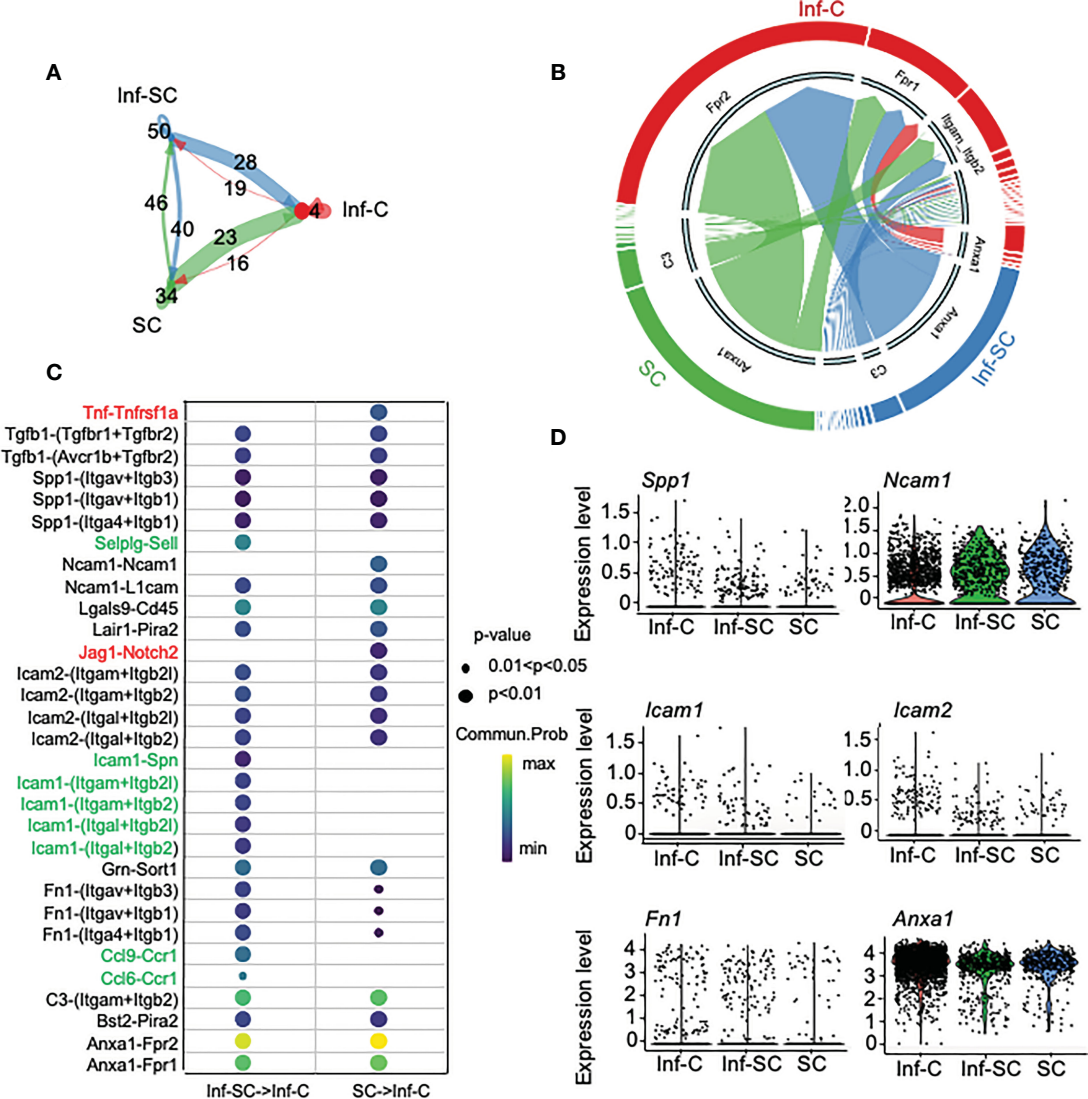
Inflammatory senescent and senescent cells regulate inflammatory cells via ligand/receptor interaction. Cellchat R software was used for predicting ligand-receptor interaction among Inf-Cs, Inf-SCs and SCs. **(A)** Circle plot showing the interaction strength predicted by Cellchat among three subclusters with interaction numbers. **(B)** Chord plot showing main ligand-receptors by cell-cell communication targeting Inf-Cs. The thickness of arrows is proportional to the interaction strength between ligand-receptor pairs. **(C)** Potential ligand-receptor interaction between Inf-SCs or SCs and Inf-Cs. Red indicates the ligand-receptor pairs detected only in Inf-SCs *vs*. Inf-Cs. Green indicates the ligand-receptor pairs detected only in SCs *vs*. Inf-Cs. Black indicates the ligand-receptor pairs detected in both Inf-SCs *vs*. Inf-Cs and SCs *vs*. Inf-Cs. Communication probability and p-values are indicated by circle size and color. **(D)** Violin plot showing the expression level of predicted ligands among Inf-Cs, Inf-SCs and SCs.

To investigate how Inf-Cs, Inf-SCs and SCs interact with each other, we first applied Cellchat analysis, a software that can predict potential ligand-receptor interactions, based on differential expression levels of ligand/receptors pairs and numbers of interactions (15). Cellchat analysis indicated that the strength of interaction was much greater when Inf-SCs and SCs were set as sender/source cells and Inf-Cs were set as recipient cells than other source-recipient pairs (**Figure 4A**), indicating that Inf-Cs are likely the target cells of Inf-SCs and SCs *via* ligand-receptor interaction. The chord diagram showed ligand-receptor pairs expressed by SCs and Inf-SCs to target Inf-Cs, and the major pairs included *Anxa*-*Fpr1*/*Fpr2* and *C3*-(*Itgam+Itgb2*) (**Figure 4B**). We found that 31 ligand-receptor pairs were expressed by Inf-SC or SC subsets to target the Inf-C subset (**Figure 4C**). Some ligands interacted with more than one receptor, including *Tgfβ1* (Transforming Growth Factor beta 1), *Spp1* (Secreted Phosphoprotein 1), *Ncam1* (Neural Cell Adhesion Molecule 1), *Icam1 and 2* (Intercellular adhesion molecule 1 and 2), and *Fn1* (Fibronectin-1). Interestingly, apart from TGFβ1, which we reported to contribute to delayed fracture healing in aged mice (3), most elevated ligand genes encode cell adhesion proteins (**Figure 4D**).

**Figure 5:**
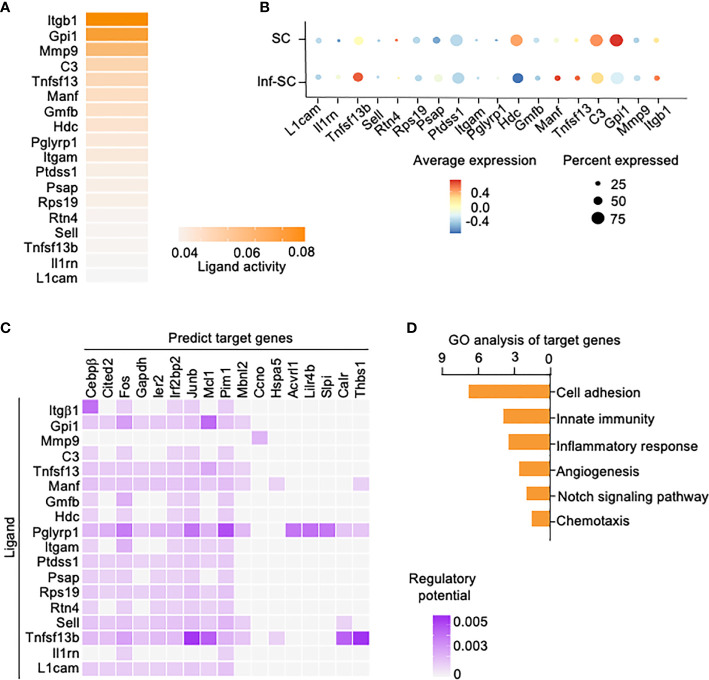
Potential ligands from inflammatory senescent and senescent cells and potential target genes in inflammatory cells. Nichenet software was used for predicting ligand expression by SCs and Inf-SCs, and target genes in Inf-Cs. **(A)** Top 18 ligands expressed by SCs and Inf-SCs. **(B)** Expression of top 18 ligands in SCs and Inf-SCs. **(C)** Ligand-target prediction between ligands from Inf-SCs and SCs and target genes in Inf-Cs. **(D)** Top 6 upregulated pathways by gene ontology analysis of predicted target genes.

We also performed Nichenet analysis, a software that can both predict ligands from sender cells and target genes from receiver cells with an intercellular communication process of interest (20). We set the Inf-SCs and SCs as sender/niche cells and Inf-Cs as receiver cells, based on the results from Cellchat (**Figure 4A**). Nichenet analysis predicted 62 ligands (**Supplemental Figure 2**). The top 18 ligands expressed by Inf-SCs and SCs are listed in **Figures 5A, B**, some of which were similar to those detected by Cellchat in **Figure 4C**, including adhesion molecules, *Itgβ1* (Integrin Subunit Beta 1), *C3* (Complement component 3), *Itgαm* (the integrin alpha M chain), *Sell* (Selectin L), and TNF family members, *Tnfsf13* (TNF Superfamily Member 13). Ligand-target gene matrix analysis detected numerous target genes under the regulation of ligands from Inf-SCs and SCs (**Figure 5C**). Some of the target genes are reported to be highly relevant to inflammatory responses, such as *Cebpβ* (CCAAT/enhancer-binding protein beta) (26, 27). GO analysis of predicted target genes from all 62 ligands revealed several biological processes related to cell adhesion, immunity and inflammation, indicating that the ligands expressed by Inf-SCs and SCs could drive inflammatory response in Inf-Cs (**Figure 5D**).

### Senescent cells promote an inflammatory phenotype in callus-derived mesenchymal progenitors

**Figure 6:**
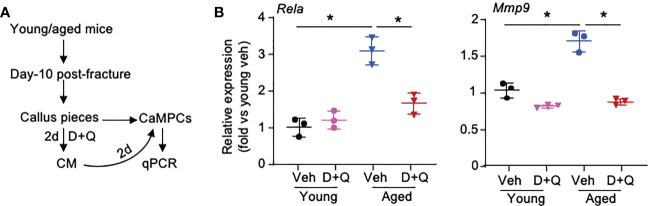
Senescent cells promote inflammatory gene expression of callus-derived mesenchymal progenitors. Young and aged mice were sacrificed at 10 dpf, and callus tissues were used to generate senescent CM. **(A)** Callus pieces were cultured in the presence of senolytic drugs, 200 nM dasatinib **(D)** + 20 μM quercetin (Q) for 2 d to generate CM. CaMPCs were treated with 30% of CM for 2 d. **(B)** Expression levels of inflammation-associated gene, *Rela*, and SASP factor, *Mmp9*, in CaMPCs examined by qPCR. Relative mRNA expression is the fold-change versus young vehicle-treated cells as 1. n=3 wells. Repeated once. Two-way ANOVA followed by Tukey *post-hoc* test. Data represent mean ± SD. *p<0.05 vehicle- versus D+Q-treated cells.

Based on the results of cell-cell commination analyses in **Figures 4** and **5**, we hypothesized that SCs and Inf-SCs produce secretory factors that have paracrine effects on callus-derived mesenchymal progenitors (CaMPCs) to induce them to develop an inflammatory phenotype, e.g. express inflammatory genes, which can be blocked by senolytic drugs. We collected conditioned medium (CM) from callus pieces that were isolated from young and aged mice in the presence and absence of the senolytic drugs, dasatinib and quercetin. The rationale for using CM from young and aged callus pieces is that we previously reported significantly increased SC numbers in callus of aged mice (3). We treated CaMPCs with CM and examined the expression of the inflammatory genes, *Rela* and *Mmp9* (**Figure 6A**). In comparison with CM from young mice, CM from aged mice increased the expression of *Rela* and *Mmp9*, which was prevented by dasatinib+quercetin (**Figure 6B**), indicating that CM from aged mice induces an inflammatory phenotype in CaMPCs by a SC-mediated mechanism.

**Figure 7:**
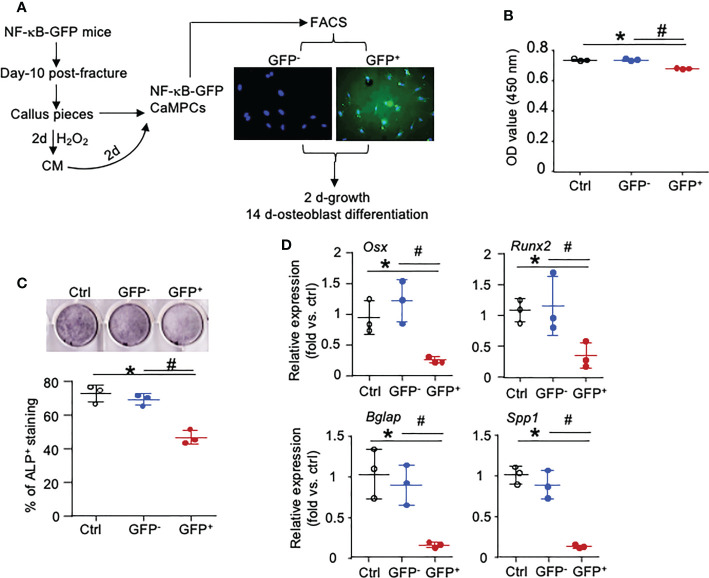
NF-κB-GFP+ callus-derived mesenchymal progenitors have reduced osteoblast function. Young NF-κB-GFP mice were sacrificed at 10 dpf, and CaMPCs were generated from callus cultures. **(A)** CaMPCs isolated from NF-κB-GFP mice were treated with senescent CM and subjected to FACS as GFP^+^ and GFP^-^ cells. Control (ctrl) group is the untreated/unsorted CaMPCs isolated from NF-κB-GFP mice, which we used as GFP negative cells for sorting GFP^-^ and GFP^+^ cells following treatment. **(B)** Cell growth assessed by a CCK8 kit. n=3 wells. Repeated once. **(C)** Osteoblast differentiation assessed by ALP staining. n=3 wells. Repeated once. **(D)** Expression levels of genes associated to osteoblast differentiation and mineralization determined by qPCR. Relative mRNA expression is the fold-change *vs*. control as 1. n=3 wells. Repeated once. Data represent mean ± SD. One-way ANOVA followed by Tukey *post-hoc* test. *p<0.05 ctrl versus GFP^+^ cells; ^#^p<0.05 GFP^+^ versus GFP^-^ cells.

To determine if Inf-Cs induced by SC and Inf-SC have decreased osteoblast differentiation capacity, we used NF-κB-GFP reporter mice that enable us to isolate cells with high NF-κB (=GFP^+^ cells) as inflammatory cells (17). We collected CM from H_2_O_2_-induced SCs, treated CaMPCs from NF-κB-GFP reporter mice, and sorted GFP^+^ and GFP^-^ cells (**Figure 7A**; **Supplemental Figure 3**). Compared to cells that were treated with CM from PBS-treated calluses, about 12% cells that were treated with CM from H_2_O_2_-treated calluses became GFP^+^ cells, indicating that NF-κB signaling was activated by factors produced by SCs (data not shown). The GFP^+^ cells had a small, but significantly reduced cell growth compared to GFP^-^ cells (**Figure 7B**). After cells were cultured in osteoblast differentiation medium for 2 weeks, GFP^+^ cells had significantly lower alkaline phosphatase (ALP) staining (**Figure 7C**) and expression levels of genes associated to both osteoblast differentiation (*Osx*, *Runx2*) and mineralization (Osteocalin-*Bglap*, Ostepontin-*Spp1*) than GFP^-^ cells (**Figure 7D**). These data indicate that inflammatory stromal cells induced by SC CM have impaired osteoblast differentiation and mineralization ability.

## Discussion

SCs produce SASPs, which include some pro-inflammatory factors produced by Inf-Cs. Thus, It is important to distinguish SCs from Inf-Cs because different drugs would be used to inhibit their adverse effects in some clinical conditions. The big discovery of this study is that Inf-Cs are transcriptionally different from SCs. In the current study, we analyzed scRNA-seq data from aged callus stromal cells and demonstrated that the cells include Inf-C, Inf-SC and SC subsets, based on their gene expression profiles. Cell-cell communication analyses predicted that SCs are the predominant cells that affect Inf-Cs. Further, our *in vitro* cell culture experiments indicated that SCs promote an inflammatory phenotype in callus mesenchymal progenitor cells, and these inflammatory callus stromal cells have reduced intrinsic osteoblast differentiation capacity. Thus, the big discovery of our study is that Inf-Cs are transcriptionally different from SCs. Our data suggest that prevention of inflammatory phenotypes in mesenchymal progenitor cells by blocking SCs may serve as an additional mechanism for senolytic therapy-enhanced fracture healing.

Why is it important to distinguish Inf-Cs from SCs? In the elderly, inflammation and senescence often co-exist due to age-associated chronic inflammation (28, 29) and age-associated cellular senescence (30, 31). However, inflammation and senescence have different causes, cellular processes, and therapies. Inflammatory processes are associated with immune responses and activation of NF-κB signaling in living organisms, including short-term acute inflammation and long-term chronic inflammation. Chronic inflammation is a low-grade unresolved immune response that occurs during aging, known as inflammaging, and is a risk factor for many aging-related diseases (32). Different from inflammation, cellular senescence is stimulated by multiple stress signals, including replicative senescence, DNA damage, programmed development senescence, and oncogene activation (33). Senescence-associated pathways are associated with activation of the cell cycle regulators, p53/p21^WAF1/CIP1^ and p16^INK4A^/pRB, and NF-κB and c/EBPβ are important downstream transcription factors (24, 34, 35). While both processes produce pro-inflammatory cytokines, Coleman et al. reported that senescent endothelial cells may have a protective role to limit local inflammatory responses (36). Indicative of the mixed nature of the cellular processes in inflammation and senescence, anti-inflammation treatment was reported to decrease senescence and increase osteogenic function in skeletal stem progenitor cells (9), and senolytic drugs had diminished anti-inflammatory effects on atherosclerotic lesions (37).

Senolytic drugs that kill SC cells have been used in 12 clinical trials for age-associated conditions, including skeletal diseases. For example, Khosla et al. are conducting a phase-2 clinical trial in healthy women over 70 years of age, in which subjects will take the senolytic drugs, dasatinib+quercetin or fisetin in a 3 day-on and 28 day-off cycle for 5 cycles. Serum bone turnover makers, C-terminal telopeptide of type I collagen (bone resorption) and A procollagen type I intact N terminal propeptide (bone formation), will be measured before and after the treatment as primary outcome measures (ClinicalTrials.gov Identifier: NCT04313634). This trial will be closed by April 2023. However, although there is no clinical trial examining the effects of senolytics in human fracture repair, recent studies reported that dasatinib+quercetin promote fracture healing in young (17) and aged mice (3).

Unlike consistent reports of beneficial effects of senolytic drugs in fracture healing (38, 39), conflicting findings have been reported for inflammatory drugs (NSAIDs) in fracture healing, despite their analgesic potency being well established (40). A systematic review of all available literature, including animal and clinical studies, reported a great diversity in the data presented in the studies (41), leaving clinicians puzzled over potential safety issues. Thus, well-randomized prospective clinical trials are warranted.

Chronic inflammation has been shown to induce senescence of mesenchymal stem/progenitor cells in fracture healing and rheumatoid arthritis (9, 42). Josephson et al. reported that mesenchymal stem/progenitor cells in middle-aged mice have an increased senescent phenotype and that NSAID treatment can improve fracture healing in middle-aged mice (9). However, emerging evidence also indicates that senescence is an upstream source of inflammatory factors (37, 43). Biavasco et al. reported that oncogene-activated senescence of human hematopoietic stem and progenitor cells promotes systemic inflammation by secreting TNFα (37, 43). In the present study, we observed that treatment of CaMPCs with SC CM only induced their inflammatory phenotype, but not cellular senescence (**Supplemental Figure 4**). We suspect that either the SASP induced by H_2_O_2_ alone was not enough to trigger senescence of CaMPCs or the concentration of SASPs in the CM was too low to induce senescence of CaMPCs.

Can we distinguish Inf-Cs from SCs since both cell types express the same SASP factors? We initially attempted to use SASP expression to distinguish Inf-Cs from SCs in our data set, such as *Tgfβ1, Tnfα, Il1a, Il1b, Il4*, and *Il6*, but failed because there is no difference in the expression between Inf-Cs and SCs. We selected cells expressing NF-κB p65 as Inf-Cs because NF-κB is a well-known inducer of inflammation (22). Under our experimental conditions, most of the NF-κB^+^ cells did not stain positively for β-galactosidase, a marker of SCs (**Supplemental Figure 4**). The finding that callus SCs also express genes associated with ROS and DNA damage can help to distinguish them from Inf-Cs.

Although it is generally accepted that inflammatory stimuli trigger cellular senescence (25, 44), the relationship between senescence and inflammation still be the “chicken or egg conundrum”. Our study demonstrated or partially demonstrated that SCs are different from Inf-Cs and SCs inflamed callus stromal cells, which caused the decreased osteogenic ability of these Inf-stromal cells. Our Cellchat analysis predicts that callus SCs and Inf-SCs affect Inf-Cs, based on the finding that SCs and Inf-SCs produce more ligands/factors than Inf-Cs (**Figure 4A**). Thus, it is possible that within a combined aging and injury micro-environment where the number of both Inf-Cs and SCs are increased, SCs likely function as effector cells to influence Inf-Cs. In addition, Nichenet analysis identified several target genes in Inf-Cs that may be regulated by ligands expressed by SCs and Inf-SCs, such as *Itgβ1*/*Cebpβ*, and *Gip1*/*Mcl1* (**Figure 5C**). C/EBPβ is a transcription factor often involved in inflammation (45, 46). Mcl1 (Myeloid Cell Leukemia 1), a member of the Bcl-2 family for maintaining cell viability (47), can act as a molecular switch for double-strand break DNA repair (48) perhaps by having an anti-senescence effect.

We also found that NF-κB^+^ CaMPCs have reduced osteoblast differentiation capacity. This is not surprising because several mechanisms by which NF-κB activation inhibits mesenchymal progenitor cell differentiation to osteoblasts have been reported. For example, conditional deletion of TRAF3 (TNF Receptor Associated Factor 3), a negative regulatory protein of NF-κB in *Prx1-expressing* mesenchymal progenitor cells, inhibited osteoblast formation by promoting degradation of β-catenin (49). Activation of the Wnt-β-catenin signaling pathway is negatively regulated by formation of a β-catenin destruction complex composed of the proteins, APC (Adenomatous polyposis coli), Annexin and GSK3β (Glycogen synthase kinase 3 β) (50). Interestingly, we found high expression of *Anxa1, Anxa2, Gsk3β, Apc* in Inf-Cs (**Supplemental Figure 5**), suggesting that the down-regulation of β-catenin signaling in inflammatory CaMPCs may be related to high expression of these negative regulators.

Limitations of the current study include the following. Since we used *Rela/Relb*-expressing cells to define Inf-Cs, it is possible that Inf-Cs are heterogenous with different biologic functions. More study is needed to investigate interactions between Inf-Cs and SCs and further sub-cluster analysis of Inf-Cs with a pro-senescent phenotype, such as pro-inflammatory factor-expressing Inf-Cs. We need to determine if these cell subsets exist *in vivo*. We currently do not have a set of surface markers for flow cytometry or immunostaining to identify these potential subsets, which also limits further determination of their function. We found Inf-SCs, cells that express genes related to both inflammation and cellular senescence. These Inf-SCs appear to have upregulated pathways similar to SCs. The origin of Inf-SCs is not clear and it is not known if they represent an intermediary state between Inf-Cs and SCs. Our trajectory analysis suggests that Inf-SCs do not originate from Inf-Cs (**Supplemental Figure 6**). Functional differences among these subsets need to be investigated. Another limitation is that we are unable to confirm our findings in patients with bone fracture, which will require high quality of scRNA-seq data from human callus cells. In our scRNAseq dataset, we did not detect the expression of genes related to chondrogenesis (*Sox9, Col2a1, Col10a1*). We suspect that the digestion method used in this study does not allow us to isolate cells with chondrogenic property from callus cells. In the future, we should modify our isolation protocol to collect cells with chondrogenic property from callus cells and investigate cellular senescence in this cell population.

In summary, callus SCs not only express SASP factors, but also genes related to ROS and DNA damage, which distinguishes them from Inf-Cs. Bioinformatic analyses predict strong interactions between Inf-Cs and SCs and potential influence of SCs on Inf-Cs by producing active ligands, although further analysis of Inf-C sub-populations is needed to elucidate the contribution of Inf-Cs to the increased cellular senescence in aged bone fracture. Despite the limitations, this study has initially answered our original question: what is the difference between inflammatory cells and SCs in aged callus: they have different gene signatures, SCs produce more factors than Inf-Cs, and SCs play a dominant role in driving inflammation and decreased osteogenic capacity in callus stromal cells.

## Data availability statement

The original contributions presented in the study are included in the article/**Supplementary Material**. Further inquiries can be directed to the corresponding author.

## Ethics statement

The animal study was reviewed and approved by the University of Rochester Committee for Animal Resources (protocol number: 2001-121R).

## Author contributions

JL, XL, AM, HZ, BB, and LX designed the study. JL, YY, and HZ performed experiments. JL, XL, AM, and YY performed bioinformatic analyses. JL and LX wrote the original manuscript. JL, XL, LX wrote the original rebuttal for resubmission. All authors contributed to the article and approved the submitted version.
