# Supplementary material for: Molecular signatures distinguish senescent cells from inflammatory cells in aged mouse callus stromal cells
Source: Front Endocrinol (Lausanne). 2023 Feb 16;14:1090049. doi: 10.3389/fendo.2023.1090049 (PMC9981154; doi:10.3389/fendo.2023.1090049)
Supplement: Supplementary file 1 [file Presentation_1.pptx]

## Slide 1
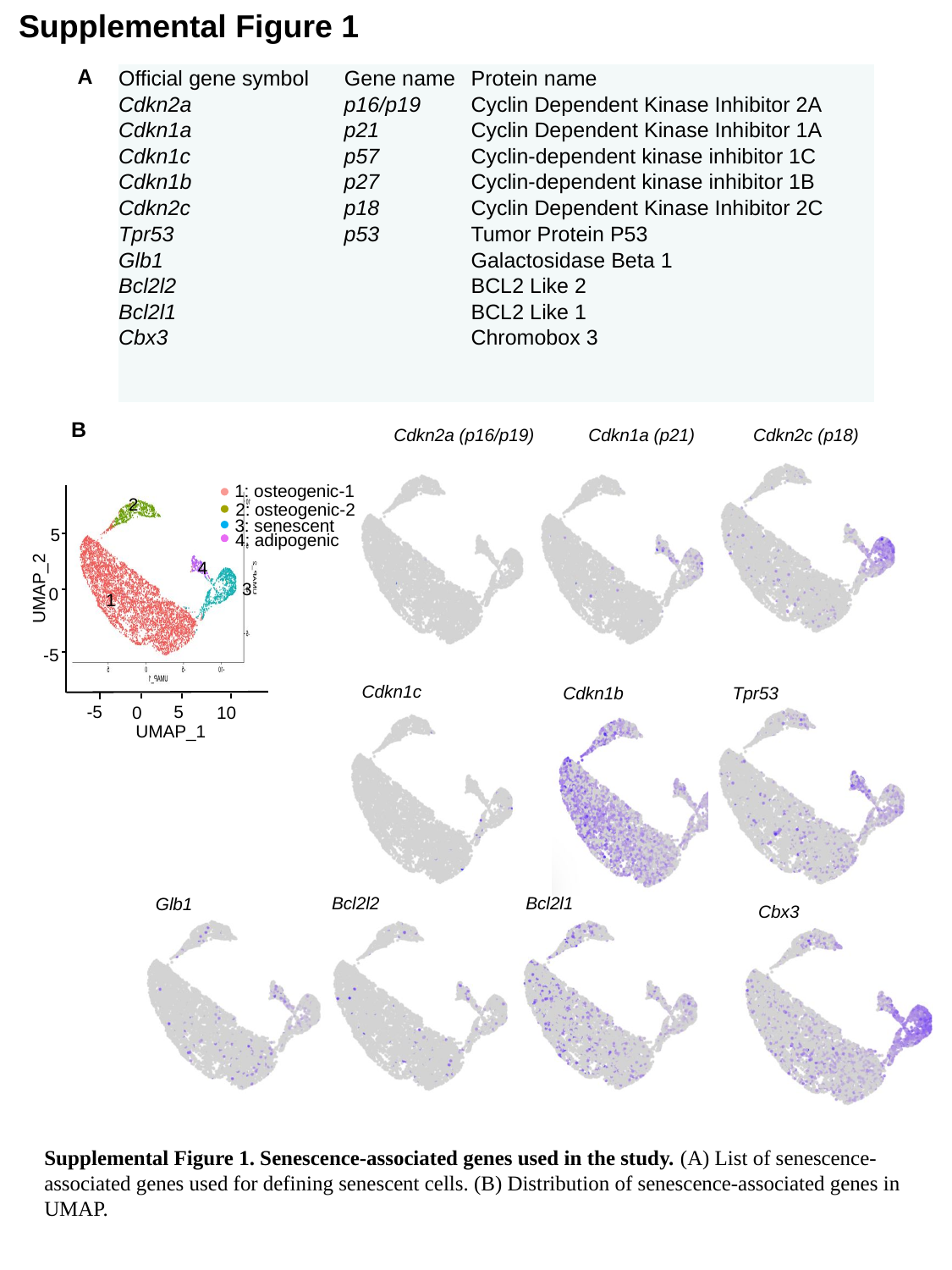

Supplemental Figure 1
A
| Official gene symbol Gene name | | Protein name |
| --- | --- | --- |
| Cdkn2a | p16/p19 | Cyclin Dependent Kinase Inhibitor 2A |
| Cdkn1a | p21 | Cyclin Dependent Kinase Inhibitor 1A |
| Cdkn1c | p57 | Cyclin-dependent kinase inhibitor 1C |
| Cdkn1b | p27 | Cyclin-dependent kinase inhibitor 1B |
| Cdkn2c | p18 | Cyclin Dependent Kinase Inhibitor 2C |
| Tpr53 | p53 | Tumor Protein P53 |
| Glb1 | | Galactosidase Beta 1 |
| Bcl2l2 | | BCL2 Like 2 |
| Bcl2l1 | | BCL2 Like 1 |
| Cbx3 | | Chromobox 3 |
| | | |
| | | |
B
Cdkn2a (p16/p19)
Cdkn1a (p21)
Cdkn2c (p18)
1: osteogenic-1
5
UMAP_2
0
-5
-5
5
10
0
2
2: osteogenic-2
3: senescent
4: adipogenic
4
3
1
Cdkn1c
Cdkn1b
Tpr53
UMAP_1
Bcl2l2
Bcl2l1
Glb1
Cbx3
Supplemental Figure 1. Senescence-associated genes used in the study. (A) List of senescence-associated genes used for defining senescent cells. (B) Distribution of senescence-associated genes in UMAP.

## Slide 2
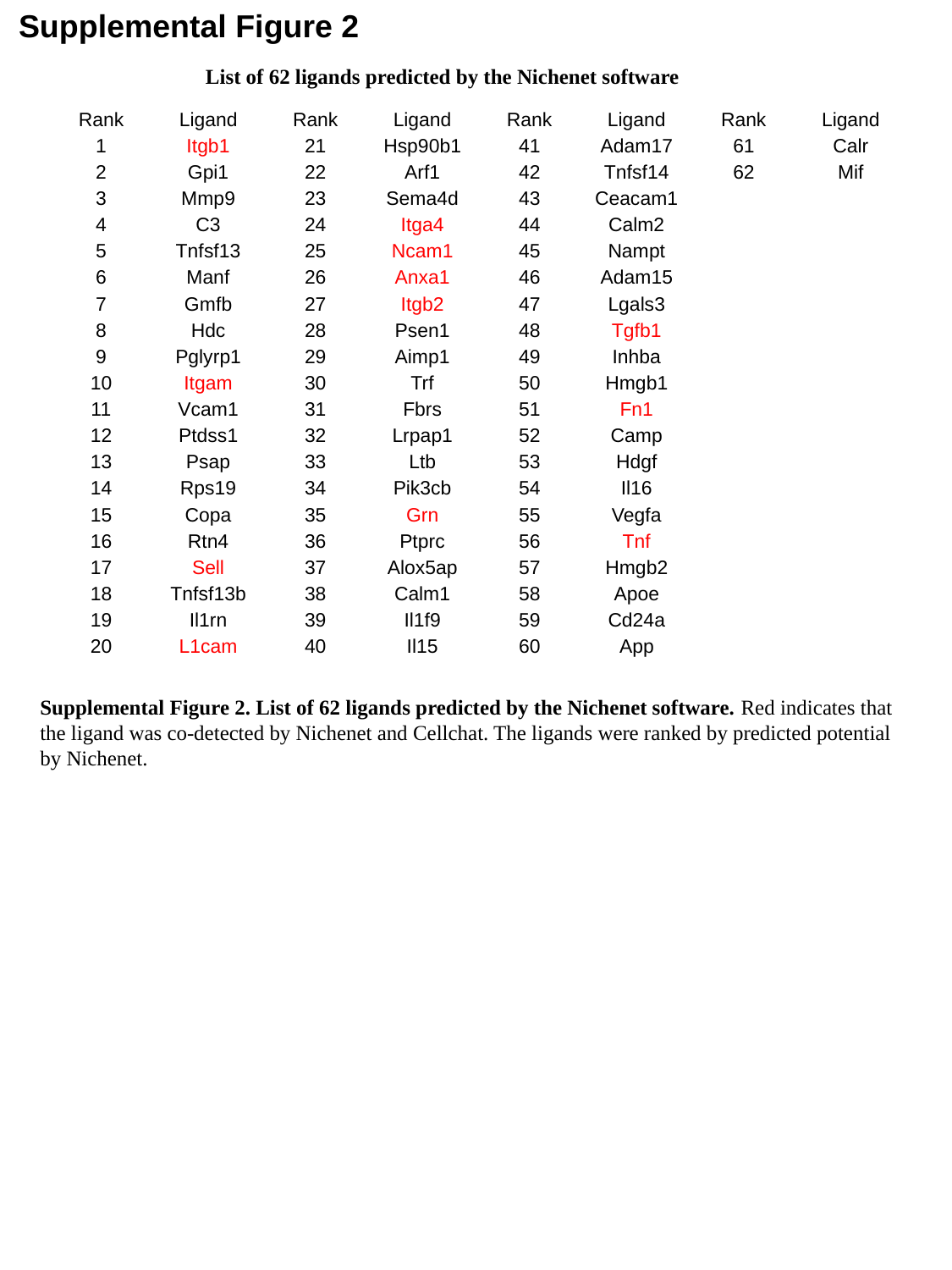

Supplemental Figure 2
 List of 62 ligands predicted by the Nichenet software
| Rank | Ligand | Rank | Ligand | Rank | Ligand | Rank | Ligand |
| --- | --- | --- | --- | --- | --- | --- | --- |
| 1 | Itgb1 | 21 | Hsp90b1 | 41 | Adam17 | 61 | Calr |
| 2 | Gpi1 | 22 | Arf1 | 42 | Tnfsf14 | 62 | Mif |
| 3 | Mmp9 | 23 | Sema4d | 43 | Ceacam1 | | |
| 4 | C3 | 24 | Itga4 | 44 | Calm2 | | |
| 5 | Tnfsf13 | 25 | Ncam1 | 45 | Nampt | | |
| 6 | Manf | 26 | Anxa1 | 46 | Adam15 | | |
| 7 | Gmfb | 27 | Itgb2 | 47 | Lgals3 | | |
| 8 | Hdc | 28 | Psen1 | 48 | Tgfb1 | | |
| 9 | Pglyrp1 | 29 | Aimp1 | 49 | Inhba | | |
| 10 | Itgam | 30 | Trf | 50 | Hmgb1 | | |
| 11 | Vcam1 | 31 | Fbrs | 51 | Fn1 | | |
| 12 | Ptdss1 | 32 | Lrpap1 | 52 | Camp | | |
| 13 | Psap | 33 | Ltb | 53 | Hdgf | | |
| 14 | Rps19 | 34 | Pik3cb | 54 | Il16 | | |
| 15 | Copa | 35 | Grn | 55 | Vegfa | | |
| 16 | Rtn4 | 36 | Ptprc | 56 | Tnf | | |
| 17 | Sell | 37 | Alox5ap | 57 | Hmgb2 | | |
| 18 | Tnfsf13b | 38 | Calm1 | 58 | Apoe | | |
| 19 | Il1rn | 39 | Il1f9 | 59 | Cd24a | | |
| 20 | L1cam | 40 | Il15 | 60 | App | | |
Supplemental Figure 2. List of 62 ligands predicted by the Nichenet software. Red indicates that
the ligand was co-detected by Nichenet and Cellchat. The ligands were ranked by predicted potential
by Nichenet.

## Slide 3
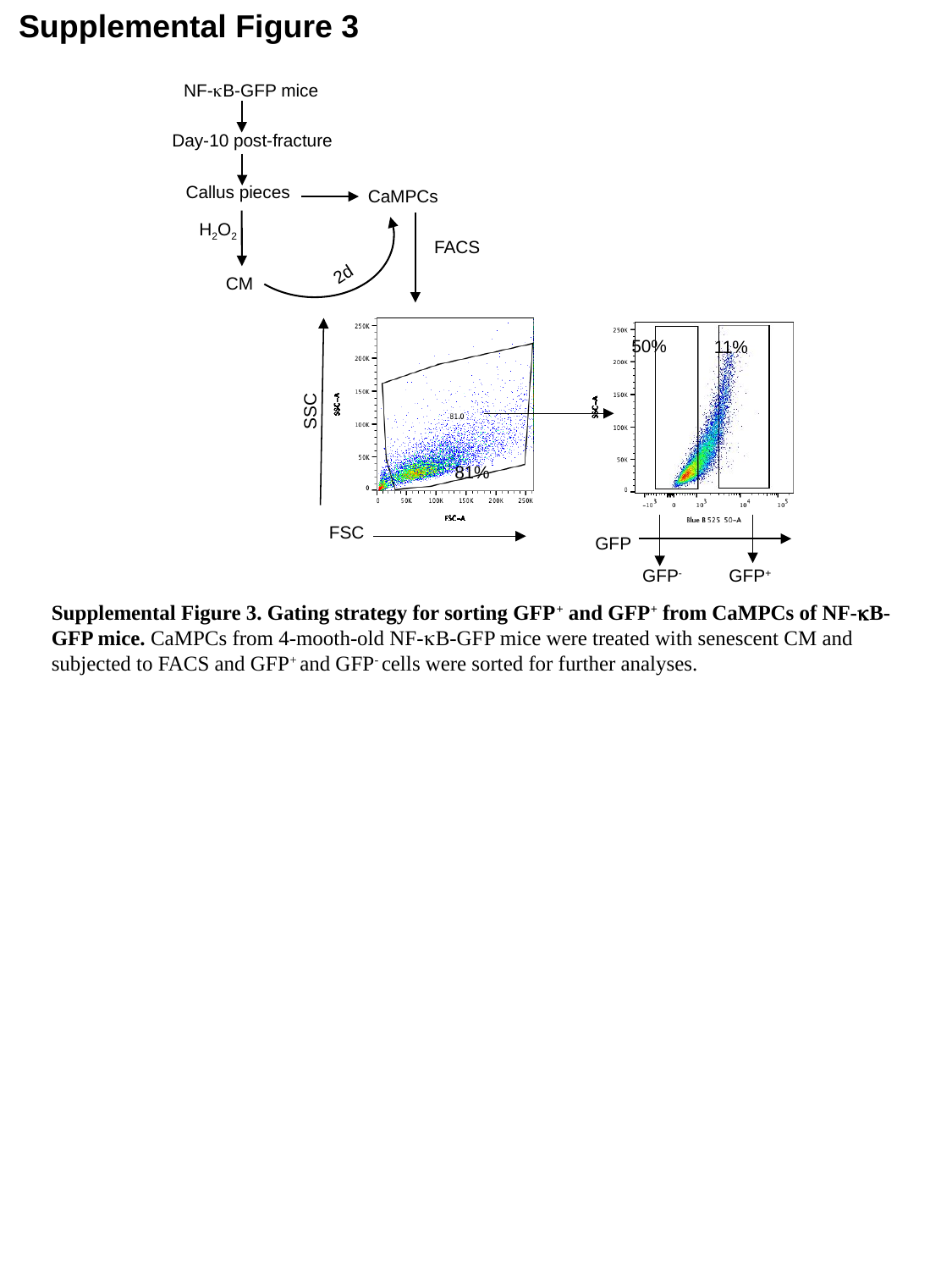

Supplemental Figure 3
NF-kB-GFP mice
Day-10 post-fracture
Callus pieces
CaMPCs
 H2O2
FACS
2d
CM
50%
11%
SSC
81%
FSC
GFP
GFP-
GFP+
Supplemental Figure 3. Gating strategy for sorting GFP+ and GFP+ from CaMPCs of NF-kB-GFP mice. CaMPCs from 4-mooth-old NF-kB-GFP mice were treated with senescent CM and subjected to FACS and GFP+ and GFP- cells were sorted for further analyses.

## Slide 4
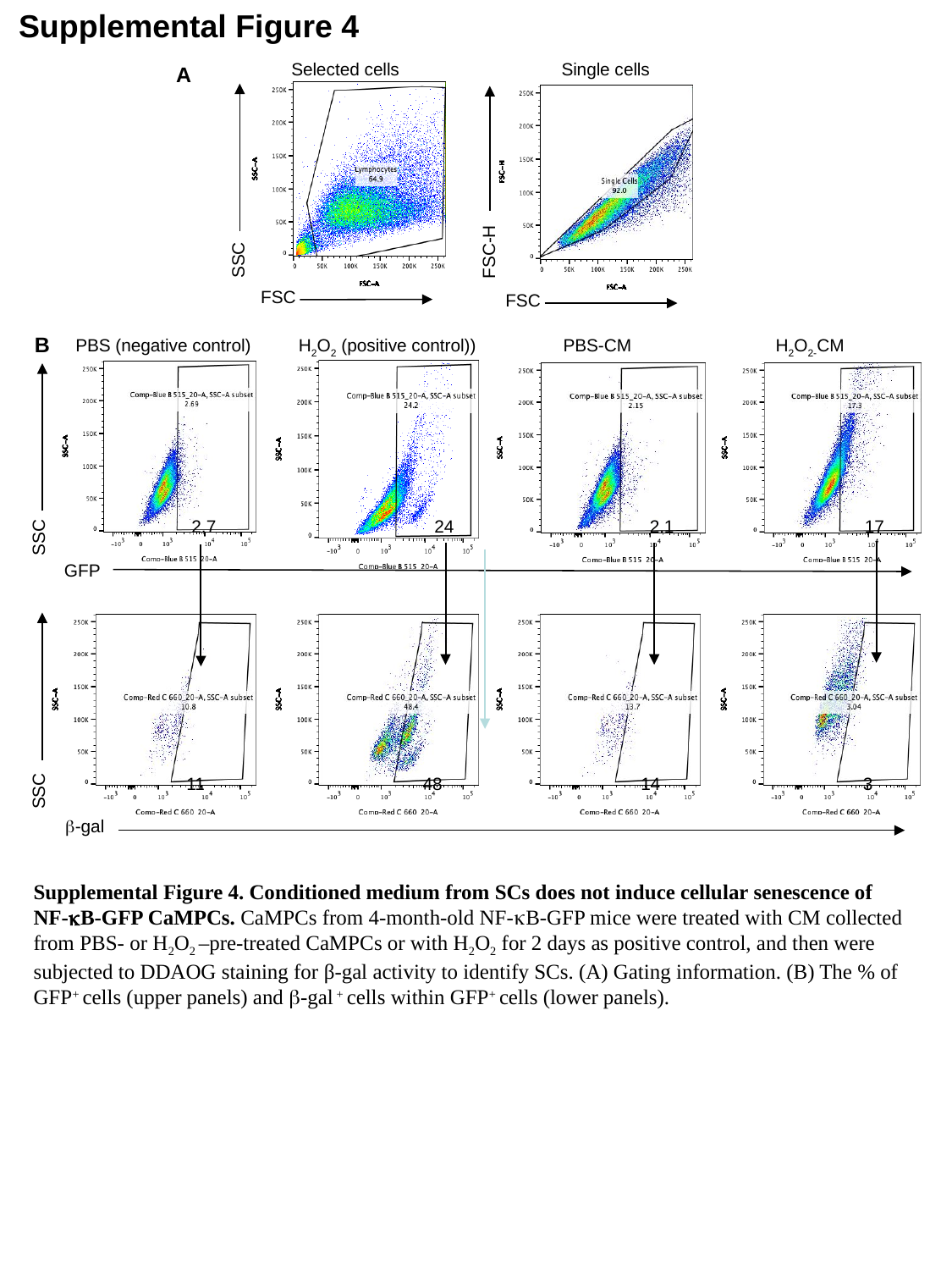

Supplemental Figure 4
Selected cells
Single cells
A
FSC-H
SSC
FSC
FSC
B
PBS (negative control)
H2O2 (positive control))
PBS-CM
H2O2-CM
SSC
2.7
24
 2.1
17
GFP
 11
 48
 14
 3
SSC
b-gal
Supplemental Figure 4. Conditioned medium from SCs does not induce cellular senescence of NF-kB-GFP CaMPCs. CaMPCs from 4-month-old NF-kB-GFP mice were treated with CM collected from PBS- or H2O2 –pre-treated CaMPCs or with H2O2 for 2 days as positive control, and then were subjected to DDAOG staining for β-gal activity to identify SCs. (A) Gating information. (B) The % of GFP+ cells (upper panels) and b-gal + cells within GFP+ cells (lower panels).

## Slide 5
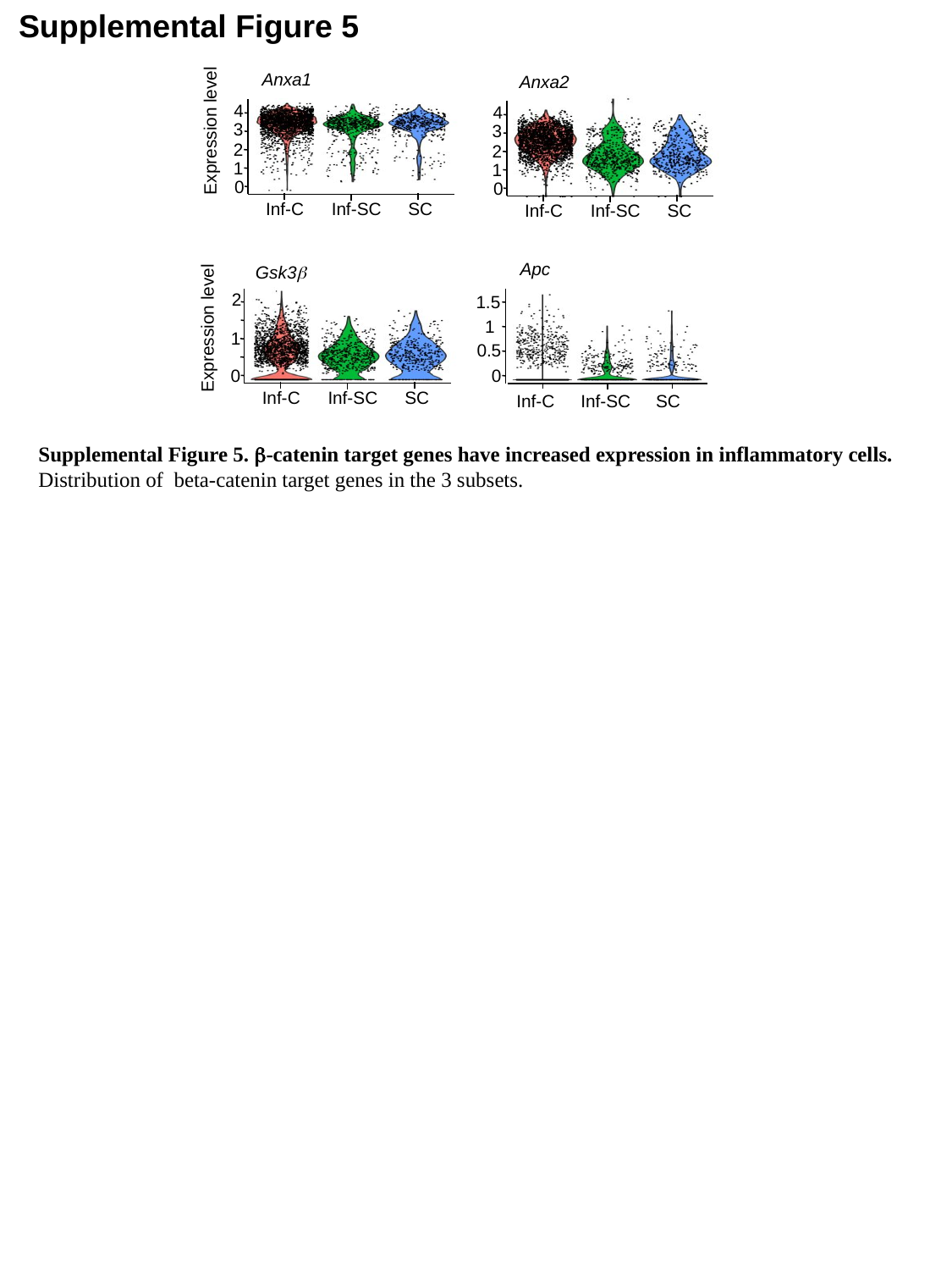

Supplemental Figure 5
Anxa1
Anxa2
4
3
2
1
0
Inf-SC
Inf-C
SC
4
3
2
1
0
Inf-SC
Inf-C
SC
Expression level
Apc
Gsk3b
2
1
0
Inf-SC
Inf-C
SC
1.5
1
0.5
0
Expression level
Inf-C
Inf-SC
SC
Supplemental Figure 5. b-catenin target genes have increased expression in inflammatory cells. Distribution of beta-catenin target genes in the 3 subsets.

## Slide 6
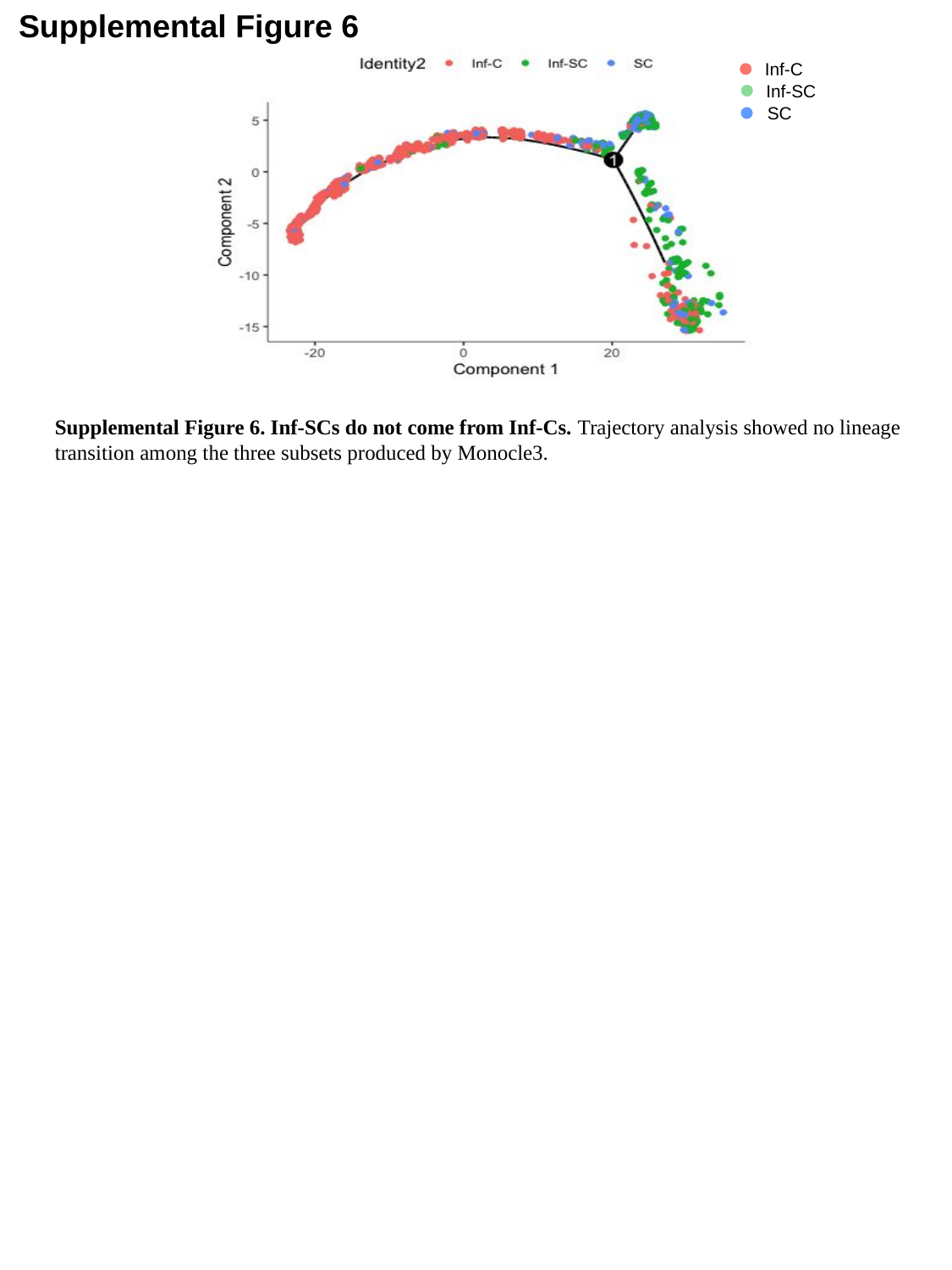

Supplemental Figure 6
Inf-C
Inf-SC
SC
Supplemental Figure 6. Inf-SCs do not come from Inf-Cs. Trajectory analysis showed no lineage transition among the three subsets produced by Monocle3.

## Slide 7
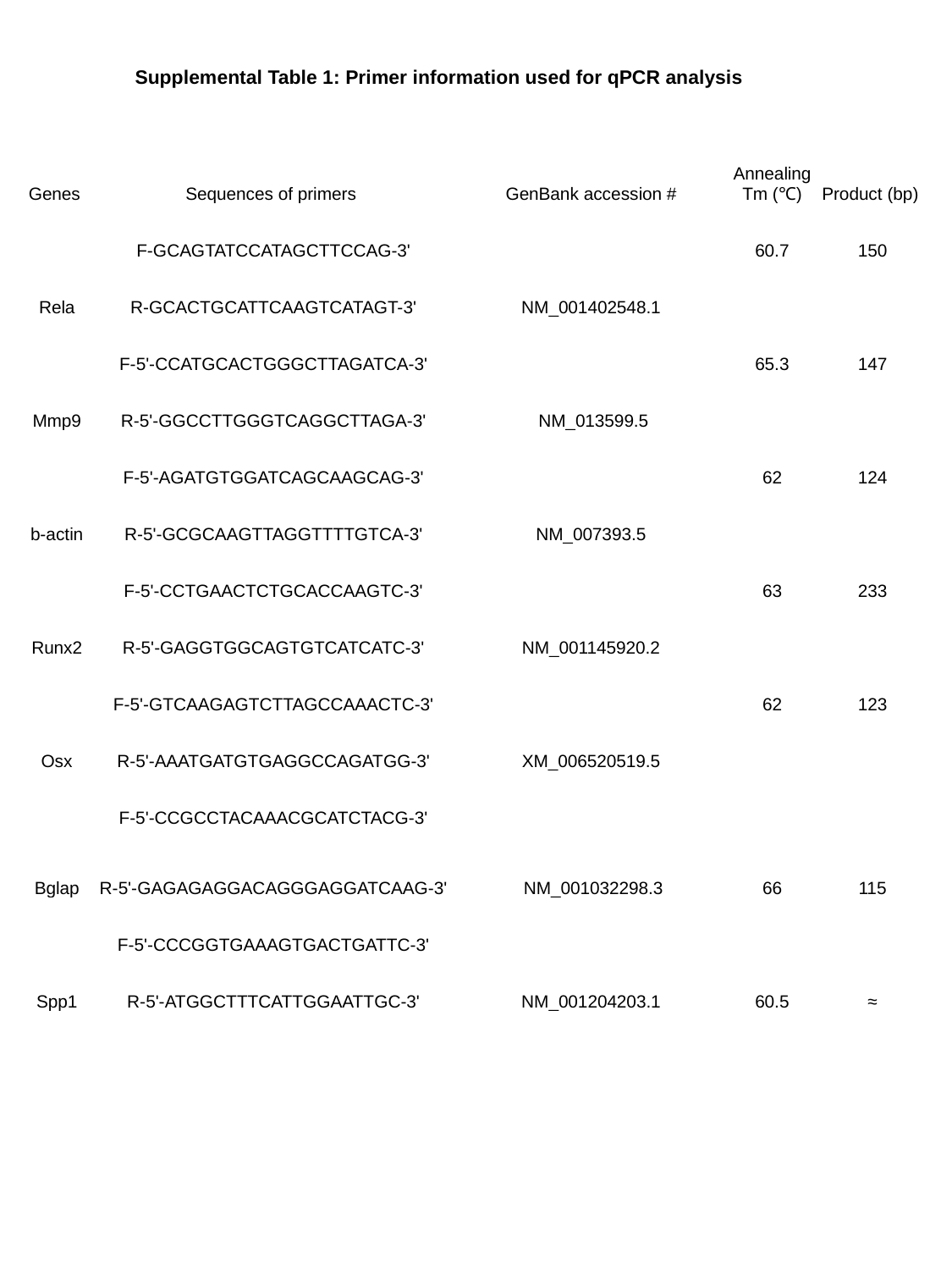

Supplemental Table 1: Primer information used for qPCR analysis
| Genes | Sequences of primers | GenBank accession # | Annealing Tm (℃) | Product (bp) |
| --- | --- | --- | --- | --- |
| Rela | F-GCAGTATCCATAGCTTCCAG-3' | NM\_001402548.1 | 60.7 | 150 |
| | R-GCACTGCATTCAAGTCATAGT-3' | | | |
| Mmp9 | F-5'-CCATGCACTGGGCTTAGATCA-3' | NM\_013599.5 | 65.3 | 147 |
| | R-5'-GGCCTTGGGTCAGGCTTAGA-3' | | | |
| b-actin | F-5'-AGATGTGGATCAGCAAGCAG-3' | NM\_007393.5 | 62 | 124 |
| | R-5'-GCGCAAGTTAGGTTTTGTCA-3' | | | |
| Runx2 | F-5'-CCTGAACTCTGCACCAAGTC-3' | NM\_001145920.2 | 63 | 233 |
| | R-5'-GAGGTGGCAGTGTCATCATC-3' | | | |
| Osx | F-5'-GTCAAGAGTCTTAGCCAAACTC-3' | XM\_006520519.5 | 62 | 123 |
| | R-5'-AAATGATGTGAGGCCAGATGG-3' | | | |
| Bglap | F-5'-CCGCCTACAAACGCATCTACG-3' | NM\_001032298.3 | 66 | 115 |
| | R-5'-GAGAGAGGACAGGGAGGATCAAG-3' | | | |
| Spp1 | F-5'-CCCGGTGAAAGTGACTGATTC-3' | NM\_001204203.1 | 60.5 | ≈ |
| | R-5'-ATGGCTTTCATTGGAATTGC-3' | | | |

## Slide 8
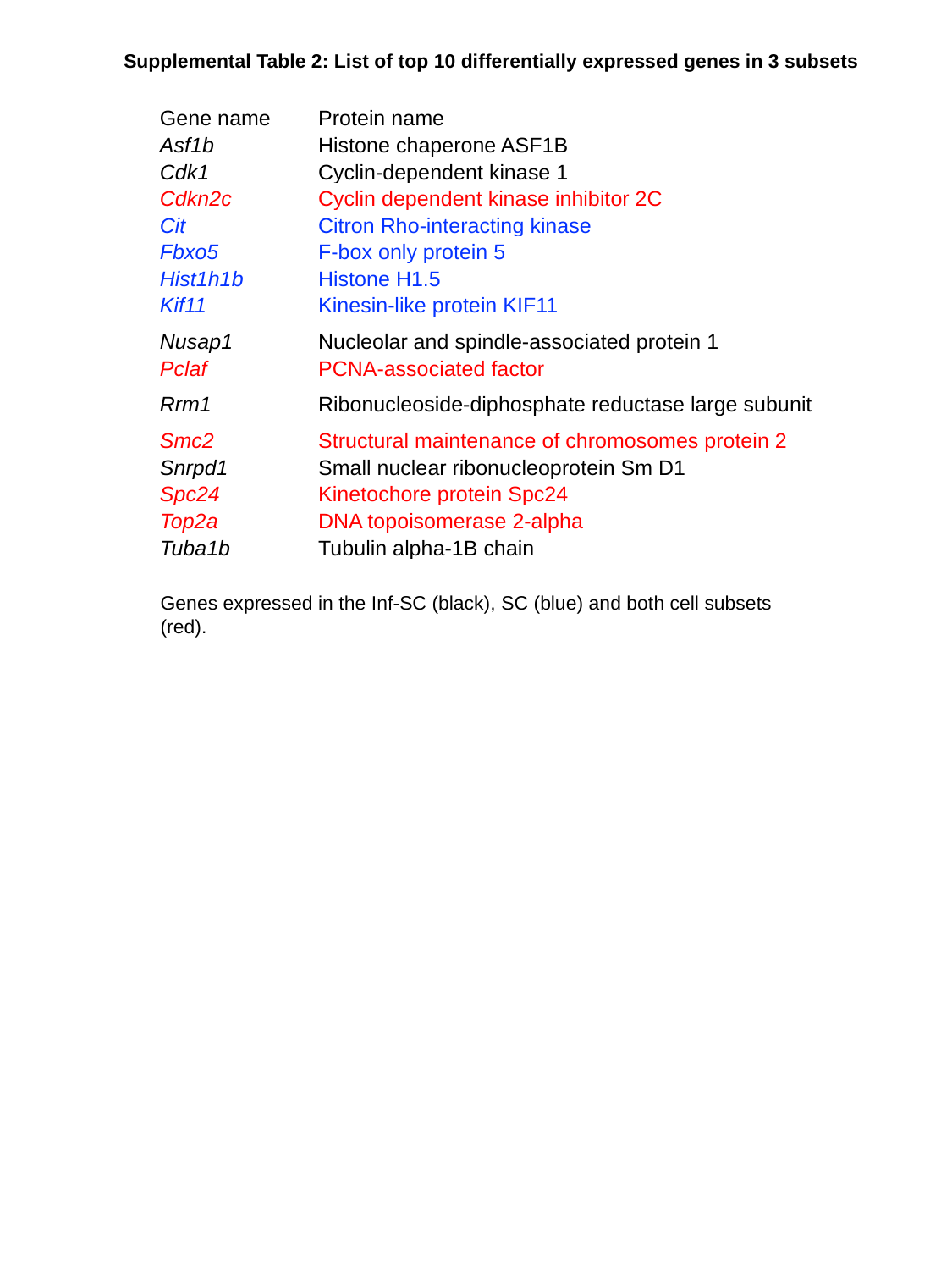

Supplemental Table 2: List of top 10 differentially expressed genes in 3 subsets
| Gene name | Protein name |
| --- | --- |
| Asf1b | Histone chaperone ASF1B |
| Cdk1 | Cyclin-dependent kinase 1 |
| Cdkn2c | Cyclin dependent kinase inhibitor 2C |
| Cit | Citron Rho-interacting kinase |
| Fbxo5 | F-box only protein 5 |
| Hist1h1b | Histone H1.5 |
| Kif11 | Kinesin-like protein KIF11 |
| Nusap1 | Nucleolar and spindle-associated protein 1 |
| Pclaf | PCNA-associated factor |
| Rrm1 | Ribonucleoside-diphosphate reductase large subunit |
| Smc2 | Structural maintenance of chromosomes protein 2 |
| Snrpd1 | Small nuclear ribonucleoprotein Sm D1 |
| Spc24 | Kinetochore protein Spc24 |
| Top2a | DNA topoisomerase 2-alpha |
| Tuba1b | Tubulin alpha-1B chain |
Genes expressed in the Inf-SC (black), SC (blue) and both cell subsets (red).
